# The effect of journal impact factor, reporting conflicts, and reporting funding sources, on standardized effect sizes in back pain trials: a systematic review and meta-regression

**DOI:** 10.1186/s12891-015-0825-6

**Published:** 2015-11-30

**Authors:** Robert Froud, Tom Bjørkli, Philip Bright, Dévan Rajendran, Rachelle Buchbinder, Martin Underwood, David Evans, Sandra Eldridge

**Affiliations:** Clinical Trials Unit, Warwick Medical School, University of Warwick, Gibbet Hill Road, Coventry, CV4 7AL UK; Norge Helsehøyskole, Campus Kristiania, Prinsens Gate 7-9, 0152, Oslo, Norway; European School of Osteopathy, The Street, ME14 3DZ Boxley, Maidstone, UK; Barts and the London School of Medicine and Dentistry, Queen Mary University of London, 58 Turner Street, London, E1 2AB Whitechapel UK; Monash Department of Clinical Epidemiology, Cabrini Institute and Department of Epidemiology and Preventive Medicine, Monash University, Suite 41, Cabrini Medical Centre, 183 Wattletree Road, Malvern, 3144, Melbourne, Victoria Australia

**Keywords:** Back pain, Impact factor, Effect size, Conflicts of interest, Funding, Reporting, Publication bias, Meta-regression, REML

## Abstract

**Background:**

Low back pain is a common and costly health complaint for which there are several moderately effective treatments. In some fields there is evidence that funder and financial conflicts are associated with trial outcomes. It is not clear whether effect sizes in back pain trials relate to journal impact factor, reporting conflicts of interest, or reporting funding.

**Methods:**

We performed a systematic review of English-language papers reporting randomised controlled trials of treatments for non-specific low back pain, published between 2006-2012. We modelled the relationship using 5-year journal impact factor, and categories of reported of conflicts of interest, and categories of reported funding (reported none and reported some, compared to not reporting these) using meta-regression, adjusting for sample size, and publication year. We also considered whether impact factor could be predicted by the direction of outcome, or trial sample size.

**Results:**

We could abstract data to calculate effect size in 99 of 146 trials that met our inclusion criteria. Effect size is not associated with impact factor, reporting of funding source, or reporting of conflicts of interest. However, explicitly reporting ‘no trial funding’ is strongly associated with larger absolute values of effect size (adjusted *β*=1.02 (95 % CI 0.44 to 1.59), *P*=0.001). Impact factor increases by 0.008 (0.004 to 0.012) per unit increase in trial sample size (*P*<0.001), but does not differ by reported direction of the LBP trial outcome (*P*=0.270).

**Conclusions:**

The absence of associations between effect size and impact factor, reporting sources of funding, and conflicts of interest reflects positively on research and publisher conduct in the field. Strong evidence of a large association between absolute magnitude of effect size and explicit reporting of ‘no funding’ suggests authors of unfunded trials are likely to report larger effect sizes, notwithstanding direction. This could relate in part to quality, resources, and/or how pragmatic a trial is.

**Electronic supplementary material:**

The online version of this article (doi:10.1186/s12891-015-0825-6) contains supplementary material, which is available to authorized users.

## Background

Low back pain (LBP) is a common and costly health complaint for which the life-time prevalence may be as high as 84 % [1]. Each year approximately 4 % of the UK population take time off work because of LBP; this equates to around 90 million working days lost and between eight and 12 million GP consultations per year [2, 3]. Globally, LBP ranks number one for contributions to Years Lived with Disability (YLDs) [4]. Several therapist-delivered interventions have been identified as useful for the early management of persistent non-specific LBP [5]. It can be difficult to choose which interventions will suit which patients, so guidelines recommend taking account of patient preference [5]. This is in part due to the different reporting methods used, the variance of reported effect sizes and a paradox that in the largest trials effect sizes tend to be quite similar irrespective of intervention and small to medium in magnitude [6, 7]. We are currently unable to determine for whom a particular treatment will be effective, as outcome has not often been shown to be dependent on participant characteristics [8]. Notwithstanding these challenges, a great deal of trust is placed in authors’ work and their estimates of treatment effect sizes, which inform decision-making and policy [9].

Readers of LBP trial reports often look first at the abstract and conclusion [9]. Some go on to look at potential known sources of bias such as lack of allocation concealment or lack of outcome assessment blinding [9]. Fewer examine funding source or conflicts of interest, possibly because it is generally assumed that, except in exceptional circumstances, these are unlikely to materially affect results. However, in 2010, an additional item recommending the reporting of trial funding was added to the CONSORT statement following the emergence of evidence that studies sponsored by pharmaceutical companies were more likely to have outcomes favouring the sponsor than studies with other sponsors, with an odds ratio of 4.05, (95 % CI 2.98 to 5.51) [10, 11]. While CONSORT does not specifically recommend reporting conflicts of interest, this is a requirement for submission to most journals. Bekelman *et al* found, in a review of reviews, that those with financial conflicts of interest were more likely to report a result in favour of pro-industry conclusions, with an odds ratio of 3.60 (95 % CI 2.63 to 4.91) [12]. Given the challenges to choosing between treatments for LBP, it is important to explore whether such an association is present in the field of LBP research. This will help to inform consumers’ interpretations of LBP trial results in the case a similarly large association exists.

Journal impact factors (IFs) quantify the average number of citations per article published in a particular journal over a specific time period; usually, the past two, or the past five-years. The Thomson Reuters Institute for Scientific Information (ISI), tracks both publications and citations of articles across journals. While use of IFs have been criticised,[13] they are widely regarded as a proxy of output quality and journal esteem, and are commonly used for advertising and self-promotion by journals on the home pages of their websites [13, 14]. Consequently, many authors select a target journal publication in-part based on that journal’s IF [15]. Effect sizes are known to become smaller as the quality of the study improves and we wanted to determine if a similar phenomenon occurred with IF, an established proxy for journal quality [16, 17]. It is not known whether the IF of a journal is associated with reported outcomes in LBP research. Submission and acceptance patterns could be influenced by several factors including perceived interest factor; perceived quality; how newsworthy a report is, especially if it is particularly novel, or goes against accepted practice in showing a null or negative result for a commonly used treatment; or how topical it is. We hypothesised that the direction or magnitude of treatment effect size is associated with journal IF.

We systematically reviewed trials of treatments for non-specific LBP to explore methodological factors as part of a larger project. In this paper, we test the null hypotheses that reported effect sizes in non-specific LBP trials are independent of (1) five-year journal IF, (2) reporting of conflicts of interest, and (3) reporting of funding sources. We also explored whether direction of outcome and trial sample size is associated with IF to find the extent to which these two factors are related to IF.

## Methods

Our focus was on trials comparing any interventions for treating non-specific LBP, measuring any patient-reported continuous (or quasi-continuous) outcome, and published over a five-year period. We included all reports of trials for interventions for non-specific LBP unless they met one or more of the following exclusion criteria: reports that self-identified as pilot/feasibility studies; trials including mixed samples of back pain (*e.g.* including neck or thoracic pain in addition to LBP); LBP due to known pathology (*e.g* cancer, ankylosing spondylitis, or disc herniation); LBP associated with pregnancy; non-English language publications; samples that included participants with radiating leg pain, or referred pain extending past the knee; and because of limited utility: non-inferiority designs (*i.e.* trials of interventions that are hypothesised to be non-different with respect to a given delta); cross-over designs; secondary reports; trials using solely objective or psychological outcome measures; and multiple publications. In the case of multiple publications, we included the first published article and excluded subsequent publications.

We searched PubMed, EMBASE, and The Cochrane Register of Controlled Trials for non-specific LBP trials published between January 1, 2006 and January 1, 2012 using the non-specific LBP string from a Cochrane Back Pain Review group search strategy [18]. Two reviewers (either TB, PB, or DR) working independently, identified all randomised controlled trial (RCT) reports for inclusion by combining all database hits in an Endnote (Version 14; Thomson Reuters, Philadelphia) library, removing duplicates, and short-listing by title and abstract. Full-texts were obtained if the titles and abstract alone contained insufficient information.

### Data abstraction

Using Microsoft Visual Basic 6.3 (Microsoft, Washington) and Microsoft Office Excel 2003 (Microsoft, Washington), we developed a front-end program to assist the data abstraction process and transfer abstracted data to a spread sheet. The program ensured consistency of data abstraction and comprehensive form completion as it insisted on correct completion of each field, producing error messages alerting reviewers to missed fields.

Two reviewers (either TB, PB, DR) independently abstracted all data. Disagreements were resolved through discussion and, if necessary, with arbitration and a fourth reviewer (RF). First the primary outcome was identified. An outcome measure was identified as ‘primary’ if (1) the outcome was nominated as the primary outcome by the authors; if no outcome was nominated, or multiple outcomes were nominated, we used (2) the outcome measure on which the sample size calculation was based; if this was not reported, we referred to (3) the first outcome measure referred to in the abstract; and if this was not identified in the abstract, we used (4) the first outcome mentioned in the paper. This approach has been used in other methodological reviews [19]. We identified the primary end-point, or used the first follow-up time point in cases when this was not clear. We then abstracted data on standardised effect size according to a set protocol.

First, we identified whether the paper reported a between-group difference in the primary outcome, or change scores or baseline and follow-up scores for each group from which we could calculate the between-group difference. The difference was recorded as positive if it favoured the intervention and negative if it favoured the control or comparison intervention. If there were more than two groups (and therefore more than two comparisons) we included the comparison with the largest effect size. To obtain the standardised effect size, if it was not directly reported, we extracted a pooled baseline SD, if available, otherwise we extracted SDs from baseline or change scores from single arms and calculated a pooled SD. We then divided the between-group difference by the SD to obtain the standardised effect size (*i.e* the standardised mean difference (SMD)) [20].

Second, we identified if the paper reported a standard error (SE) for the between-group difference. As the SE relates to the standard deviation of a specific outcome measure, and since we were looking at different outcome measures, we needed to re-standardise the SE across all outcome measures. We divided the SE by the SD extracted or calculated in the previous step to produce a re-standardised SE (*SSE*_*α*_). If SE for between-group difference was not reported, we identified whether a 95 % CI was reported for the between-group difference, and under the assumption of normality, we calculated the standardised SE according to Eq. 1 (*SSE*_*β*_). We prioritised these first two methods respectively where they were available, as these represent the adjusted errors in cases where authors included covariates within modelling. If neither the SE nor CI for a between-group difference was reported, then we estimated the crude standardised SE for the between-group difference (*SSE*_*Δ*_). As the outcomes in these LBP trials are patient reported and quasi-continuous in nature, the SE is calculated as $\frac {\sigma }{\sqrt {n}}$ in each arm. On standardising this by the SD the expression simplifies to that shown in Eq. 2.

(1) $$ SSE_{\beta}=\frac{Upp-Lwr}{2\times 1.96\times \sigma_{b}}  $$

(2) $$ SSE_{\Delta}=\frac{1}{\sqrt{n_{t}}}+\frac{1}{\sqrt{n_{c}}}  $$

*where Upp=the upper limit of the 95% CI, and Lwr= the lower limit of the 95% CI; n=sample size, t=treatment group, c=control/comparator group,σ*_*b*_*=the pooled baseline SD.*

If, during any of these steps, the required information could not be ascertained, we recorded the reason and did not analyse data from that paper. For consistency in this process we developed a flow chart for reviewers’ use (Additional file 1).

In addition, we abstracted data on the number of participants on whom data were analysed, COIs, classifying these as either ‘not reported’, ‘none reported’, or ‘some reported’; and funding status, which we classified in the same way. We used 2011 five-year IF for the corresponding journals, which we obtained from Thompson Reuters ISI Web of Knowledge (Thompson Reuters, Philadelphia).

### Analysis

We tested the null hypotheses that effect size is independent of (1) 5-year journal IF; (2) COI reporting category; and (3) funding reporting category. As high-magnitude departures from the null value regardless of direction of effect size might be equally attractive to some journals, we also explored relationships to the absolute value of effect size. We explored sample size by COI and funding reporting category compared to not reporting any details, whether sample size was independent of journal IF, and whether there was any difference in journal IF in journals publishing positive and negative trial reports.

We fitted random effects meta-regression models of effect size on IF, COI category, and funding status, using within-study SSEs (*vide supra*) to weight observations. We used a restricted maximum likelihood (REML) estimator for between-study variance [21–23]. Within the REML algorithm used, coefficients as well as between-study variance was estimated with weighted least squares, so error terms were unbiased by heteroschedasticity [24, 25]. In the case that a journal did not have an official IF, we imputed an IF value of zero. We performed sensitivity analyses, using log-transformed IF (involving small non-zero imputations for journals with no official impact factor) and sample size, to allay concerns that readers more familiar with transformations than REML estimation to overcome heteroschedasticity might have in relation to the heteroschedasticity.

We first fitted crude models, *i.e.* with only the outcome and predictor variable for each hypothesis, and subsequently fitted adjusted models including the other predictor variables; *i.e* IF, COI category, and funding category, as well as trial sample size, and publication year, in case relationships changed over time. If predictors appeared non-linear we explored fitting polynomial terms to the model.

Model fits were assessed by graphical examination of standardised predicted random effects. We did not dismiss models on the basis of a high proportion of residual error explained by heterogeneity (*i.e.I*^2^), since our focus was on the associations between effect size and characteristics across a number of different interventions and not on estimation of any one intervention effect in particular. All analyses were performed using Stata, version 12.1 for Unix (Statacorp, Texas) and we used the package *metareg* v2.6.1 to fit the meta-regression models [24, 26].

## Results

**Fig. 1:**
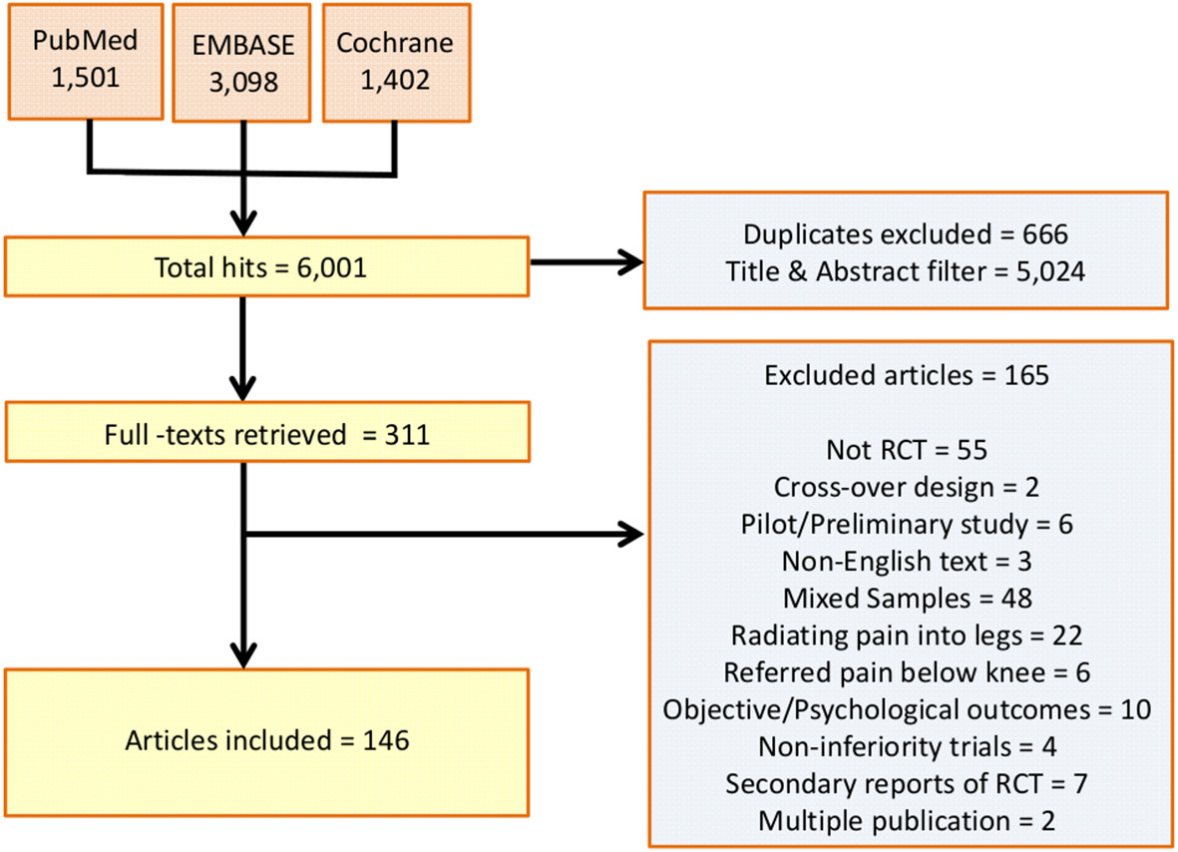
Flow chart of excluded and included trials. The figure shows the path and number of excluded and included trials

Figure 1 details hits obtained from the databases searched, exclusions made at the title and abstract phase, and 165 rejections at full-text level [27–191].

We could abstract the required effect size data on 99 trials [192–290] from the 146 articles that met inclusion criteria [192–337]. We could not abstract data on SD for the remaining 47 cases (Additional file 1). (Additional file 2: Table S1) shows the characteristics of included studies and (Additional file 3: Table S2) shows the characteristics of excluded studies.

Within our sample, the mean standardised effect size was 0.374, the mean total sample size was 149.3 (SD=217.4), ranging from 12 to 1,261, and the mean IF was 3.14 (4.33), ranging from less than 1 to 33.79. We were able to abstract the SE of the between-group difference directly in two trials (2 %), we calculated the SE from the 95 % CI of the between-group difference (Eq. 1) in 27 trials (27 %), and we calculated a the SE using Eq. 2 for the remaining 70 trials (71 %). Authors explicitly identified their primary outcome in 53 (54 %) trials. Table 1 shows that no comment on funding was made in 35 (35 %) trials, whereas authors specifically reported that there was no funding in 11 (11 %) trials, and acknowledged a funder in 53 (54 %) trials. No comments were made about COIs in the reports of 70 trials (71 %), authors explicitly stated there were no COIs in 22 trials (22 %), and reported at least one COI in 7 trials (7 %). We imputed zeros for IFs for 16 cases in which 13 unique journals did not have an official ISI IF. Table 1 also shows mean sample size, effect size, absolute effect size, and IF by each of the funding and COI categories. Compared to not reporting any funding details, sample size in trials reporting some funding was larger; absolute effect size in trials reporting no funding was larger; and IF in trials reporting some funding was higher.

**Table 1:** Mean sample size, effect size (ES), absolute effect size, and IF, by categories of funding and reported COIs

		N (%)	Sample size (SD)	ES (mean)	abES (mean)	IF (mean)
COI						
	None reported	70 (71)	149.9 (247.0)	0.389	0.67	3.144
	Reported none	22 (22)	126.5 (177.1)	0.291	0.78	3.458
	Reported some	7 (7)	214.6 (125.1)	0.409	0.41	6.001
Funding						
	None reported	35 (35)	61.8 (31.5)	0.324	0.53	2.004
	Reported none	11 (11)	87.8 (42.4)	0.499	1.43 ^∗∗∗^	2.155
	Reported some	53 (54)	219.9 (277.6) ^∗∗∗^	0.371	0.62	4.102 ^∗^

^∗^ *P*<0.05 (compared to none reported)

^∗∗∗^ *P*=0.001 (compared to none reported)

Adjusted for trial sample size, there was no evidence of a difference in journal IF between positive or negative trials (*P*=0.270). Adjusted for direction of effect, there was very strong evidence of a linear association between IF and sample size, suggesting that the IF of the publishing journal increases by 0.008 (95 % CI 0.004 to 0.012) per unit increase in total sample size (*P*<0.0005).

**Fig. 2:**
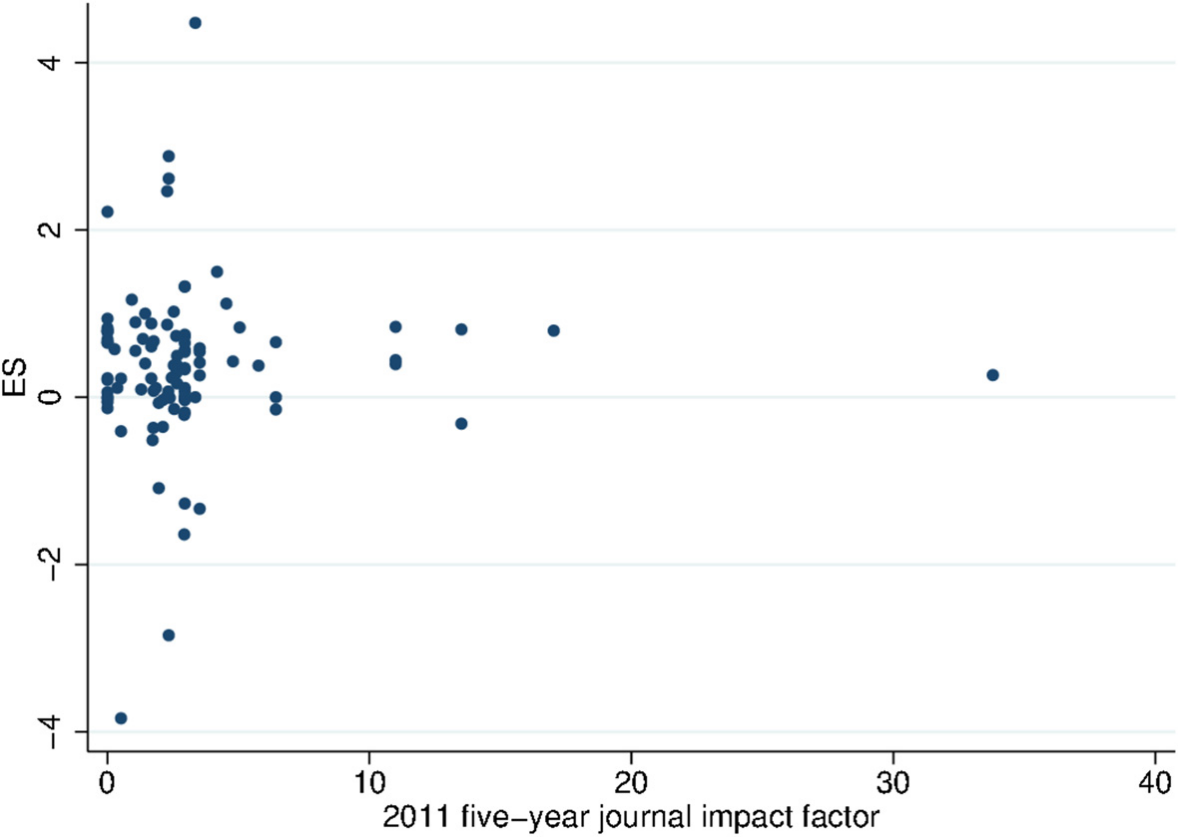
Effect size by 2011 5-year journal impact factor. The figure shows the effect size and the variance of effect size by journal impact factor

There was no evidence of an association (either linear or non-linear) between effect size and IF (*P*=0.527) but journals with low IFs tended to report trials with a wider range of effect sizes than high If journals (Fig. 2). Compared to nothing being reported about COIs, there was no evidence of an effect of reporting no COIs (*P*=0.624) or some COIs (*P*=0.950). Compared to nothing being reported about funding, there was no evidence of an effect of reporting no funding (*P*=0.481) or some funding (*P*=0.847). Table 2 shows full results, including both crude and adjusted beta estimates, and covariates.

**Table 2:** Meta-regression: the effect of impact factor, COI, Funding category, year, and sample size on effect size

		Unadjusted			Adjusted ^*b*^		
		*β*	*P*	95 % CI for *β*	*β*	*P*	95 % CI for *β*
Impact factor							
		0.01	0.81	−0.04 to 0.05	0.02	0.53	−0.03 to 0.06
COI	Category ^*a*^						
	Reported none	−0.08	0.73	−0.55 to 0.39	−0.14	0.62	−0.68 to 0.41
	Reported some	0.03	0.94	−0.70 to 0.76	0.02	0.95	−0.75 to 0.80
Funding	Category ^*a*^						
	Reported none	0.19	0.59	−0.49 to 0.86	0.27	0.48	−0.48 to 1.02
	Reported some	0.03	0.89	−0.39 to 0.45	0.05	0.85	−0.43 to 0.52
Publication year							
		−0.05	0.42	−0.16 to 0.07	−0.05	0.44	−0.17 to 0.08
Sample size							
		−0.0003	0.45	−0.001 to 0.0005	−0.0005	0.31	−0.002 to 0.0005

^*a*^comparison: none reported

^*b*^for other variables in the model

Table 3 shows the results of the meta-regression of absolute magnitudes of effect size on COI categories and funding categories, including covariates. There was no evidence of an effect of IF on absolute effect size (*P*=0.806). Compared to nothing being reported about COIs, there was no evidence of an effect of reporting no COIs (*P*=0.512) or some COIs (*P*=0.464). There was very strong evidence of a large effect of reporting no funding compared to reporting no details about funding (*β*=1.02,*P*=0.001), and no evidence of an effect of reporting some funding compared to reporting no details about funding (*P*=0.506).

**Table 3:** Meta-regression: the effect of impact factor, COI, Funding category, year, and sample size and on absolute values of effect size

		Unadjusted			Adjusted ^*b*^		
		*β*	*P*	95 % CI for *β*	*β*	*P*	95 % CI for *β*
Impact factor							
		−0.01	0.54	−0.04 to 0.02	0.004	0.81	−0.03 to 0.04
COI	Category ^*a*^						
	Reported none	0.13	0.52	−0.25 to 0.51	-0.14	0.51	−0.55 to 0.28
	Reported some	−0.24	0.42	−0.82 to 0.34	-0.21	0.46	−0.78 to 0.36
Funding	Category ^*a*^						
	Reported none	0.94	0.001	0.42 to 1.45	1.02	0.001	0.44 to 1.59
	Reported some	0.05	0.76	−0.27 to 0.37	0.12	0.51	−0.24 to 0.48
Publication year							
		−0.05	0.31	−0.14 to 0.05	−0.05	0.31	−0.14 to 0.05
Sample size							
		−0.0004	0.20	−0.001 to 0.0002	−0.0004	0.20	−0.001 to 0.0003

^*a*^comparison: none reported

^*b*^for other variables in the model

As anticipated, residual variance due to heterogeneity was high (91.06 % >*I*^2^>85.76 %) across all models. Quadratic terms in IF and sample size were not significant in either unadjusted or adjusted models. Graphical inspection of standardised predicted random effects showed adequate model fits. Sensitivity analyses (not reported) showed near identical results.

## Discussion

### Main findings, implications and comparisons to existing research

While no associations were found between effect size and IF, reporting sources of funding, or conflicts of interest, there was strong evidence of a large association between absolute magnitude of effect size and the explicit reporting of ‘no funding’. We first discuss IF and then COIs and funding.

#### Impact factor

The results show no evidence that IF is associated with effect size reported in LBP trials. Effect size is much more variable in journals with low IFs and since journals with higher IFs tend to publish larger trials this likely explains the relationship between effect size variance and journal IF. Journal IF was not associated with direction of result, although there was some evidence that trials reporting a funder had a higher IF than those who did not report funding status.

Suñé *et al* reviewed clinical trials evaluating drug therapy published between 1997 and 2004 and classified the outcomes of these trials as positive, negative, or descriptive (non-controlled) [338]. They found no difference in IF based on trial direction, but they found the IF was significantly lower in trials classified as descriptive. Littner *et al* found that over a five-year period in the field of neonatology, articles with negative results were more likely than articles with positive results to be published in journals with lower IFs [339]. Penel and Adenis found the same pattern of association in phase II trials investigating anticancer therapies [340]. Outside of the medical fields, Murtaugh has explored the relationship between standardised effects and IFs in published meta-analyses of terrestrial plant competition, predation in streams, woody plant growth under elevated *CO*_2_, and marine nutrient enrichment experiments. Using raw data, he similarly applied weighted least squares regression analysis of study-specific means of the absolute values of the log response ratios on log of journal IFs and found some evidence that in two of the four areas studied (Nutrient enrichment experiments and predation in streams) that journal IF was associated with reported effects [341].

The presence or absence of associations differs across different research areas. It may be that there is less competition in high-impact journals in terms of newsworthiness of LBP trial results relative to other fields. As it is rare for individual treatments for LBP to stand out dramatically from others in terms of effect size, effect sizes in LBP trials may not be a big driver of an acceptance decision in higher-impact journals.

#### COI and funding status

We found no evidence that COI category or reported funding status is associated with effect size reported in LBP trials. However, we observed that absolute magnitudes of effect sizes tended to be about one SD larger for trials that declared no funding compared with trials that did not report funding status. The observed association is not due to confounding by sample size. Jacob Cohen, who originally defined standardised effect sizes, considered effect sizes of 0.2 or less to be small, 0.5 to be medium, and 0.8 and above to be large [20]. Using Cohen’s categorisation, the effect size in the larger trials of interventions for LBP tend to be only small-to-medium in magnitude [7].

This relationship is in marked contrast to that observed in other fields, where evidence suggests industry-funded, industry-linked studies, or studies with an industry-funded author, report greater effect sizes than independently funded studies [11, 12, 342, 343]. In the authors’ experiences, LBP research trials tend to be more commonly funded by government and charitable organisations rather than by industry. It may be that, in the case of LBP trials, reporting larger effect sizes, may be higher amongst studies with fewer resources.

Our *a priori* approach was to compare categories of explicitly reporting no funding/COIs, and explicitly reporting funding/COIs with not reporting anything about funding/COIs, and it is these results that are reported. As a *post hoc* comparison to explore reporting of funding further, we compared trials that explicitly reported having funding with trials that explicitly reported not having funding, and found strong evidence of a large effect (*β*=−0.89 (95 % CI −1.46 to −0.33), P = 0.002), suggesting that those reporting receiving funding, report considerably smaller effect sizes than those reporting their trials were not funded.

Trial quality may partially explain the results. It has been previously shown that larger trials in non-specific LBP tend to be higher quality [17, 344]. We did not explore trial quality in our study. Another consideration may be that pragmatic trials tend to be done more often in LBP research, since many interventions under assessment are complex in nature and pharmacological interventions (which are usually of efficacy rather than effectiveness) [345] are comparatively rare. It may be that trials more toward the pragmatic end of the spectrum, which may be more difficult to do in the absence of funding given their typical requirement to be large in scale, may be associated with smaller effect sizes simply because the comparator is often another active intervention. Conversely, efficacy trials may have higher effect sizes in part due to more commonly utilising placebo/sham comparisons. We did not explicitly set out to explore this. However, as another *post hoc* comparison we looked at the intervention comparisons in our included trials, and those that were compared to sham/placebo had an effect size of 0.74 (large), in contrast with those compared to a non-sham/placebo interventions, which had an effect size of 0.29 (P = 0.077; *i.e* weak evidence of a small-to-moderate difference).

### Strengths and limitations

Meta-regression modelling is most useful in this case as a tool to assess the role of chance in the observed results. We caution against use for prediction, since epistemologically this may not be entirely sensible: prediction may involve a reversal of the direction of causality; authors likely choose journals on the basis of publishing work they believe to be newsworthy, high-quality, or of interest to a particular journal’s readership. More robust and simpler solutions to establishing the role of chance, and whether relationships between effect size and IF are monotonic could be used (such as non-parametric correlation) but, as Murtaugh points out, such approaches are less able to incorporate study-specific weights and are ultimately less powerful [341]. Also, such approaches are not as conducive to the inclusion of covariates. In this study we had sufficient power to detect a medium-to-large effect size in terms of funding category, but not in terms of COI, which were only reported in 7 % of trials.

COIs disclosure may or may not be insisted upon by a journal, or COI forms may have been completed but not reported with the article. Additionally, disclosed COIs may or may not be relevant to the trial. We explored only the presence or absence of such statements reported with the article and did not judge the relevance of disclosed COIs to the trial, nor whether the publishing journal required disclosure, and this as a limitation of our study.

The large *I*^2^ values for the models suggests that the residual variance explained by heterogeneity is very high. This is to be expected since the included trials featured many different interventions. In our analysis, other than having a detrimental effect on power, the high *I*^2^ is inconsequential to interpretation and does not present a limitation as it would in a meta-analysis of a specific treatment effect. We were not focused on estimating the effect of a specific intervention, but the association between effect size and IF, COIs, and funding across many different interventions for nsLBP, some of which will naturally have larger effects than others.

We imputed zero values for IF in the case of journals without an official IF. Many journals use unofficial IFs and including these could have been used to introduce more information into our models. We reasoned that the majority of journals without official IFs would likely have unofficial IFs of less than 1.00 and preferred to use only official values. We note that if IFs had been associated with effect size then our estimates may have been exaggerated. As we did not find any association with effect size, imputing values where there was no official IF was of limited consequence and does not affect conclusions.

In attempting to explain our results, we have hypothesised that there may be a link to study quality, which we did not explore. While there is some evidence of a small effect of poor quality on effect size in LBP trials from other work, we would welcome future investigations using the Cochrane Risk of Bias tool, since the judgement criteria in this tool can be applied to either pragmatic or efficacy trials without prejudice. Lower quality trials may have been associated with both absence of definition of primary outcome measure, where we would have used outcome measure selection method 3 or 4 (see Methods section), as well as with larger study effects. While we recorded and reported authors explicitly identifying an outcome as primary, for the trials in which this was not explicitly identified, we did not record how often primary outcome identification method 2, 3, or 4 needed to be used. So we caution that there may have been an unmeasured confounding factor.

We rejected trials from which we could not abstract population-specific SD data required for the meta-analysis. This resulted in 47 rejections and opens a possibility for bias, in the case that not reporting these data is also associated with reported effect size. While not specifically an item in the CONSORT statement, this is something one might reasonably expect to be discussed within a sample size calculation. For this reason, we suggest the absence of its reporting, is more likely to be associated with lower quality. Assuming this, and the premise that lower quality trials report larger effect sizes notwithstanding direction, are both correct, then our results will tend toward being conservative.

Finally, we restricted our systematic review to three large databases, reasoning that these index the majority of nsLBP RCTs. We acknowledge however, that the review of the period is unlikely to be exhaustive and that there may be further associations between trials indexed in other databases alone, and quality; and thus with the potential to alter results. However, our results cover most of the field and therefore provide a useful account of behaviour.

### Recommendations

Based on our results we recommend that journal editors consider giving increased scrutiny at peer-review stage to unfunded LBP trials. Researchers need to carefully consider whether the trial in question can be adequately and appropriately conducted in the absence of funding, and whether the protocol should be subject to peer review. Consumers of LBP trial reports should note this relationship in the case trials are unfunded.

The causal pathway for the relationship between funding and effect size needs further exploration. If larger effect sizes yielded by unfunded trials are incorrect, these may add noise to data consumed by review work decreasing the precision of meta-analyses. If internal validity is a factor, one might raise the question of whether it is ethically justified to undertake unfunded trials of interventions for LBP. If the extent of the pragmatism of a trial is a driving factor, then absorption of the higher absolute effect sizes into specific review work is less of a concern, but a scale of pragmatism might aid interpretation of effect size from individual trials and be useful to reviewers.

Research into the relationship between funding status and effect size, and IF and effect size, appears to be dependent of the field of research and the nature of interventions under investigation. For this reason we suggest that investigations are conducted across different fields and interventions so that the relationships between COIs and funding and effect size can be better understood and consumers can take this into consideration, as appropriate.

Authors of LBP trials should explicitly report whether or not funding was attained, as only around two-thirds of all authors are currently doing this. Moreover, more authors need to be explicit about whether or not there were COIs as in our dataset only 29 % of authors are doing this. Journals and editors could consider taking steps to ensuring this information is reported.

## Conclusions

While there is no evidence that reported funding status and reported conflicts of interest influence effect size, there is very strong evidence that authors who explicitly report that their LBP trial was unfunded tend to report larger absolute magnitudes of effect size. Journal editors, researchers, and consumers may have need to be cautious of large effect sizes in unfunded trials, possibly giving additional scrutiny to internal validity. Our results contrast with findings in pharmacological research and suggests relationships may vary by field. Further discipline-specific investigations would inform interpretation of trial reports and help identify causal pathways of associations between effect sizes and trial/report characteristics.

## Additional files

Additional file 1
**Reviewers’ flowchart.** A copy of the flowchart used by reviewers, guiding how to abstract standardised effect sizes and standardised SEs in PNG format. (PNG 198 kb)

Additional file 2
**Characteristics of included studies.** A table of characteristics of included studies in PDF format. (PDF 60 kb)

Additional file 3
**Characteristics of excluded studies.** A table of characteristics of excluded studies in PDF format. (PDF 69 kb)

## Abbreviations

CI: Confidence interval
CONSORT: Consolidated standards of reporting trials
COI: Conflict of interest
IF: Impact factor
ISI: (Thomson Reuters) Institute for scientific information
LBP: Low back pain
RCT: Randomised controlled trial
REML: Restricted maximum likelihood
SD: Standard deviation
SE: Standard error
SMD: Standardised mean difference
SSE: Re-standardised standard error
YLD: Years lived with disability
