# Supplementary figures and images for: The effect of journal impact factor, reporting conflicts, and reporting funding sources, on standardized effect sizes in back pain trials: a systematic review and meta-regression
Source: BMC Musculoskelet Disord. 2015 Nov 30;16:370. doi: 10.1186/s12891-015-0825-6 (PMC4663726; doi:10.1186/s12891-015-0825-6)

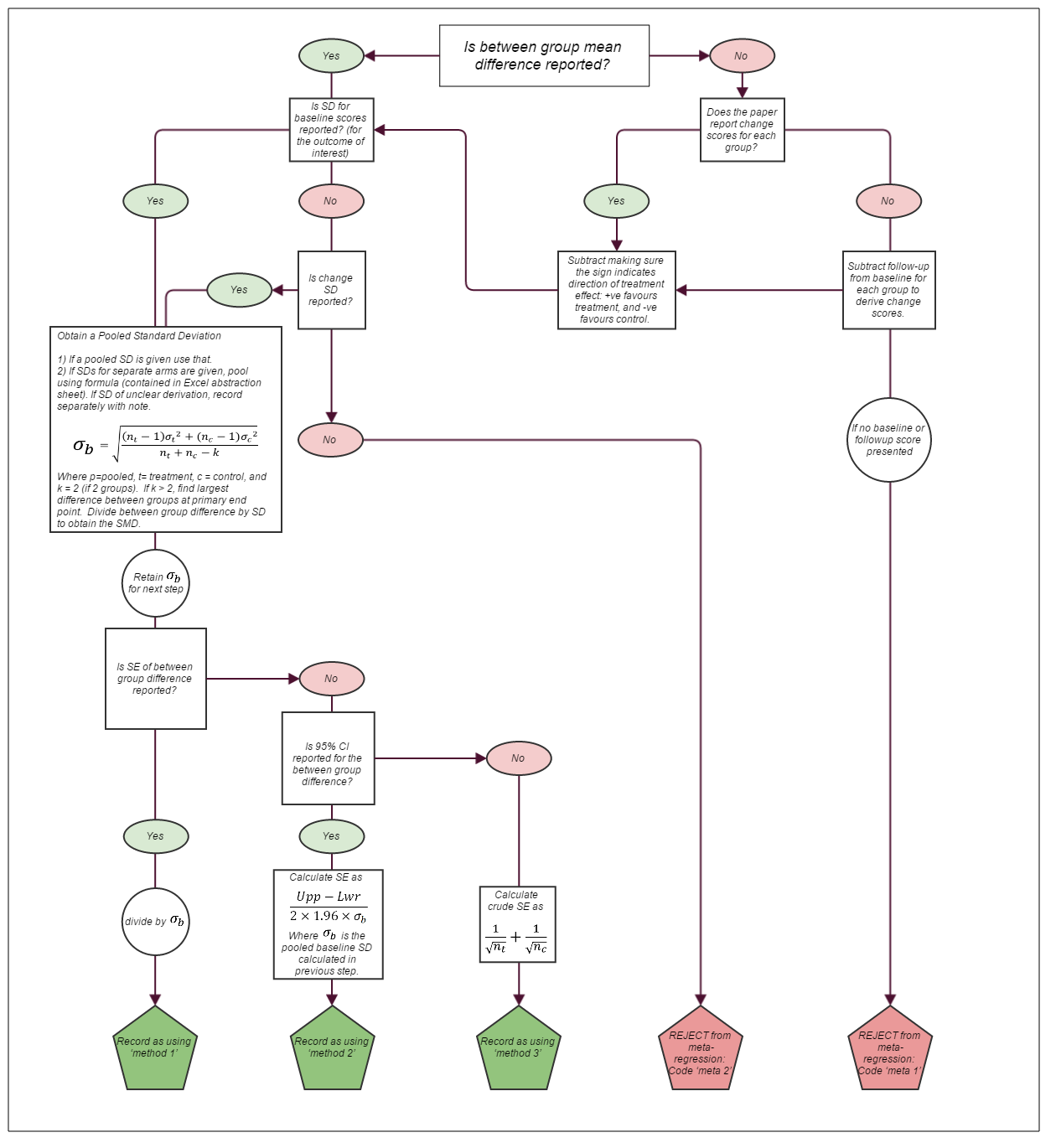

Supplement: Additional file 1 — Reviewers’ flowchart. A copy of the flowchart used by reviewers, guiding how to abstract standardised effect sizes and standardised SEs in PNG format. (PNG 198 kb) [file 12891_2015_825_MOESM1_ESM.png]
