# Supplementary material for: The effect of journal impact factor, reporting conflicts, and reporting funding sources, on standardized effect sizes in back pain trials: a systematic review and meta-regression
Source: BMC Musculoskelet Disord. 2015 Nov 30;16:370. doi: 10.1186/s12891-015-0825-6 (PMC4663726; doi:10.1186/s12891-015-0825-6)
Supplement: Additional file 2 — Characteristics of included studies. A table of characteristics of included studies in PDF format. (PDF 60 kb) [file 12891_2015_825_MOESM2_ESM.pdf]

Table S1: Included study characteristics

Lead author	Publication year	Title	Sample size	Extracted
Ackerman, W	2008	Pain Relief With Intraarticular or Medial Branch Nerve Blocks in Patients With Positive Lumbar Facet Joint SPECT Imaging: A 12-Week Outcome Study	46	Yes
Ahmed, S	2009	Evaluation of the effects of shortwave diathermy in patients with chronic low back pain	97	Yes
Akbari, A	2008	The effect of motor control exercise versus general exercise on lumbar local stabilizing muscles thickness: Randomized controlled trial of patients with chronic low back pain	49	Yes
Albaladejo, C	2010	The Efficacy of a Short Education Program and a Short Physiotherapy Program for Treating Low Back Pain in Primary Care	348	No
Anema, J	2007	Multidisciplinary Rehabilitation for Subacute Low Back Pain: Graded Activity or Workplace Intervention or Both?	112	No
Attanayake, A	2010	Clinical evaluation of selected yogic procedures in individuals with low back pain	12	No
Becker, A.	2008	Effects of Two Guideline Implementation Strategies on Patient Outcomes in Primary Care	1261	Yes
Bello, A	2010	Hydrotherapy versus land-based exercises in the management of chronic low back pain: a comparative study	12	Yes
Ben Salah Frih, Z	2009	Efficacy and treatment compliance of a home-based rehabilitation programme for chronic low back pain: A randomized, controlled study	107	Yes
Bialosky, Joel E	2009	Spinal Manipulative Therapy Has an Immediate Effect on Thermal Pain Sensitivity in People With Low Back Pain: A Randomized Controlled Trial	36	Yes
Bicalho, E	2010	Immediate effects of a high-velocity spine manipulation in paraspinal muscles activity of nonspecific chronic low-back pain subjects	40	Yes
Birkenmaier, C	2007	Medial branch blocks versus pericapsular blocks in selecting patients for percutaneous cryodenervation of lumbar facet joints	26	No
Bishop, P	2010	The Chiropractic Hospital-based Interventions Research Outcomes (CHIRO) Study: a randomized controlled trial on the effectiveness of clinical practice guidelines in the medical and chiropractic management of patients with acute mechanical low back pain	72	No
Brennan G	2006	Identifying subgroups of patients with acute/subacute ”nonspecific” low back pain	123	Yes
Brinkhaus B	2006	Acupuncture in patients with chronic low back pain	284	No

Lead author	Publication year	Title	Sample size	Extracted
Cairns M	2006	Randomized controlled trial of specific spinal stabilization exercises and conventional physiotherapy for recurrent low back pain	97	No
Calmels, P	2009	Effectiveness of a Lumbar Belt in Subacute Low Back Pain An Open, Multicentric, and Randomized Clinical Study	190	Yes
Cecchi, F	2010	Spinal manipulation compared with back school and with individually delivered physiotherapy for the treatment of chronic low back pain: a randomized trial with one-year follow-up	205	Yes
Chan, C	2011	Aerobic exercise training in addition to conventional physiotherapy for chronic low back pain: A randomized controlled trial.	43	Yes
Chang, S	2008	Effects of piroxicam-beta-cyclodextrin sachets on abnormal postural sway in patients with chronic low back pain	42	Yes
Chatzitheodorou, D	2008	The effect of exercise on adrenocortical responsiveness of patients with chronic low back pain, controlled for psychological strain.	61	Yes
Cherkin, D	2011	A Comparison of the Effects of 2 Types of Massage and Usual Care on Chronic Low Back Pain	380	Yes
Cherkin, D	2009	A Randomized Trial Comparing Acupuncture, Simulated Acupuncture, and Usual Care for Chronic Low Back Pain	606	Yes
Chiu, C	2011	The efficacy and safety of intramuscular injections of methylcobalamin in patients with chronic nonspecific low back pain: a randomised controlled trial	58	Yes
Chown, M	2008	A prospective study of patients with chronic back pain randomised to group exercise, physiotherapy or osteopathy	98	Yes
Cleland, J	2009	Comparison of the Effectiveness of Three Manual Physical Therapy Techniques in a Subgroup of Patients With Low Back Pain Who Satisfy a Clinical Prediction Rule	108	No
Costa, L	2009	Motor Control Exercise for Chronic Low Back Pain: A Randomized Placebo-Controlled Trial	154	Yes
Cuesta-Vargas, A	2011	Exercise, Manual Therapy, and Education with or Without High-Intensity Deep-Water Running for Nonspecific Chronic Low Back Pain	46	Yes
da Fonseca, J	2009	Laboratory Gait Analysis in Patients With Low Back Pain Before and After a Pilates Intervention	28	Yes
da Silva, A	2010	Evaluation of an Extract of Brazilian Arnica (Solidago chilensis Meyen, Asteraceae) in Treating Lumbago	20	No

Lead author	Publication year	Title	Sample size	Extracted
Demirel, R	2008	Effects of balneotherapy with exercise in patients with low back pain	44	Yes
Demoulin, C	2010	Effectiveness of a semi-intensive multidisciplinary outpatient rehabilitation program in chronic low back pain	160	No
Diaz Arribas, M	2009	Effectiveness of the Physical Therapy Godelive Denys-Struyf Method for Nonspecific Low Back Pain	126	Yes
Di Cesare, A	2011	Comparison between the effects of trigger point mesotherapy versus acupuncture points mesotherapy in the treatment of chronic low back pain: A short term randomized controlled trial	61	Yes
Djavid, G	2007	In chronic low back pain, low level laser therapy combined with exercise is more beneficial than exercise alone in the long term: a randomised trial	53	No
Donzelli, S	2006	Two different techniques in the rehabilitation treatment of low back pain: A randomized controlled trial	43	No
Dufour, N	2010	Treatment of Chronic Low Back Pain A Randomized, Clinical Trial Comparing Group-Based Multidisciplinary Biopsychosocial Rehabilitation and Intensive Individual Therapist-Assisted Back Muscle Strengthening Exercises	272	Yes
Dundar, U	2009	Clinical Effectiveness of Aquatic Exercise to Treat Chronic Low Back Pain	65	Yes
Durmus, D	2010	Effects of therapeutic ultrasound and electrical stimulation program on pain, trunk muscle strength, disability, walking performance, quality of life, and depression in patients with low back pain: a randomized-controlled trial	59	Yes
Durmus, D	2010	Effects of Therapeutic Ultrasound on Pain, Disability, Walking Performance, Quality of Life, and Depression in Patients with Chronic Low Back Pain: A Randomized, Placebo Controlled Trial	42	No
Durmus, D	2009	Effects of electrical stimulation program on trunk muscle strength, functional capacity, quality of life, and depression in the patients with low back pain: a randomized controlled trial	43	Yes
Engbert, K	2011	The Effects of Therapeutic Climbing in Patients with Chronic Low Back Pain	23	Yes
Ergun, H	2010	The efficacy, safety, and pharmacokinetics of intramuscular and oral phenyramidol in patients with low back pain in an emergency department	72	No
Ewert, T	2009	The Comparative Effectiveness of a Multimodal Program Versus Exercise Alone for the Secondary Prevention of Chronic Low Back Pain and Disability	183	Yes

Lead author	Publication year	Title	Sample size	Extracted
Farhadi, K	2009	The effectiveness of wet-cupping for nonspecific low back pain in Iran: A randomized controlled trial	98	Yes
Ferreira, M.	2007	Comparison of general exercise, motor control exercise and spinal manipulative therapy for chronic low back pain: A randomized trial	224	Yes
Ferreira, M	2009	Relationship between spinal stiffness and outcome in patients with chronic low back pain	191	Yes
Field, T	2007	Lower back pain and sleep disturbance are reduced following massage therapy	30	Yes
Fiore, P	2011	Short-term effects of high-intensity laser therapy versus ultrasound therapy in the treatment of low back pain: a randomized controlled trial	30	No
Franca, F	2010	Segmental stabilization and muscular strengthening in chronic low back pain - a comparative study	30	Yes
Friedrich, M	2009	Long-Term Effect of a Combined Exercise and Motivational Program on the Level of Disability of Patients With Chronic Low Back Pain	74	No
Frost H	2006	Randomized controlled trial of physiotherapy compared with advice for low back pain	200	No
Gladwell, V	2006	Does a Program of Pilates Improve Chronic Low Back Pain.	34	Yes
Glazov, G	2009	Laser acupuncture for chronic non-specific low back pain: a controlled clinical trial	90	No
Gohner, W	2006	Preventing chronic back pain: evaluation of a theory-based cognitive-behavioural training programme for patients with subacute back pain	47	Yes
Goldby L	2006	A randomized controlled trial investigating the efficiency of musculoskeletal physiotherapy on chronic low back disorder	200	Yes
Haake, M	2007	German Acupuncture Trials (GERAC) for Chronic Low Back Pain	1117	Yes
Hagen, E	2010	Adding a physical exercise programme to brief intervention for low back pain patients did not increase return to work	246	No
Hall, A	2011	Tai Chi Exercise for Treatment of Pain and Disability in People With Persistent Low Back Pain: A Randomized Controlled Trial	160	Yes
Hancock, M	2007	Assessment of diclofenac or spinal manipulative therapy, or both, in addition to recommended fi rst-line treatment for acute low back pain: a randomised controlled trial	235	No

Lead author	Publication year	Title	Sample size	Extracted
Harts, C	2008	A high-intensity lumbar extensor strengthening program is little better than a low-intensity program or a waiting list control group for chronic low back pain: a randomised clinical trial	59	Yes
Hartvigsen, J	2010	Supervised and non-supervised Nordic walking in the treatment of chronic low back pain: a single blind randomized clinical trial	136	Yes
Henchoz, Y	2010	Role of Physical Exercise in Low Back Pain Rehabilitation A Randomized Controlled Trial of a Three-Month Exercise Program in Patients Who Have Completed Multidisciplinary Rehabilitation	103	Yes
Henchoz, Y	2010	Functional multidisciplinary rehabilitation versus outpatient physiotherapy for non-specific low back pain: randomised controlled trial	92	Yes
Heymans M	2006	The effectiveness of high-intensity versus low-intensity back schools in an occupational setting	299	Yes
Hondras, M	2009	A randomized controlled trial comparing 2 types of spinal manipulation and minimal conservative medical care for adults 55 years and older with subacute or chronic low back pain	213	No
Hsieh, L	2006	Treatment of low back pain by acupuncture and physical therapy	110	Yes
Iles, R	2011	Telephone coaching can increase activity levels for people with non-chronic low back pain: a randomised trial	26	Yes
Inoue, M	2006	Relief of low back pain immediately after acupuncture treatment - a randomised, placebo controlled trial	31	Yes
Johnson, R	2007	Active exercise, education, and cognitive behavioral therapy for persistent disabling low back pain: a randomized controlled trial	223	Yes
Juni, P	2009	A randomised controlled trial of spinal manipulative therapy in acute low back pain	104	No
Kapitza, K	2010	First Non-Contingent Respiratory Biofeedback Placebo versus Contingent Biofeedback in Patients with Chronic Low Back Pain: A Randomized, Controlled, Double-Blind Trial	42	Yes
Kell, R	2011	The response of persons with chronic nonspecific low back pain to three different volumes of periodized musculoskeletal rehabilitation	240	Yes
Kell, R	2009	A comparison of two forms of periodized exercise rehabilitation programs in the management of chronic nonspecific low-back pain	27	Yes

Lead author	Publication year	Title	Sample size	Extracted
Kofotolis N	2006	Effects of two 4-week proprioceptive neuromuscular facilitation programs on muscle endurance, flexibility, and functional performance in women with chronic low back pain	86	Yes
Koldas Dogan, S	2008	Comparison of three different approaches in the treatment of chronic low back pain	55	Yes
Kroll,H	2008	A randomized, double-blind, prospective study comparing the efficacy of continuous versus pulsed radiofrequency in the treatment of lumbar facet syndrome	26	Yes
Kulisch, A	2009	Effect of thermal water and adjunctive electrotherapy on chronic low back pain: A double blind, randomized, folow up study.	71	No
Kumar, S	2009	Efficacy of two multimodal treatments on physical strength of occupationally subgrouped male with low back pain	102	Yes
Kumar, S	2010	Comparative efficacy of two multimodal treatments on male and female sub-groups with low back pain (part II)	141	No
Kumar, S	2009	Efficacyof dynamic muscular stabilization techniques (DMST) over conventional techniques in rehabilitation of chronic low back pain.	30	Yes
Lalanne, K	2009	Modulation of the flexion-relaxation response by spinal manipulative therapy: a control group study	27	Yes
Lamb, S	2010	Group cognitive behavioural treatment for low-back pain in primary care: a randomised controlled trial and cost-effectiveness analysis	598	Yes
Lambeek, L	2010	Randomised controlled trial of integrated care to reduce disability from chronic low back pain in working and private life	134	No
Lau, P	2008	Early physiotherapy intervention in an Accident and Emergency Department reduces pain and improves satisfaction for patients with acute low back pain: a randomised trial	102	Yes
Lengsfeld, M	2007	Passive rotary dynamic sitting at the workplace by office-workers with lumbar pain: a randomized multicenter study	231	Yes
Leonhardt, C	2007	TTM-based motivational counselling does not increase physical activity of low back pain patients in a primary care setting - A cluster-randomized controlled trial	1261	Yes
Lewis, C	2011	Strain-Counterstrain therapy combined with exercise is not more effective than exercise alone on pain and disability in people with acute low back pain: a randomised trial	85	Yes

Lead author	Publication year	Title	Sample size	Extracted
Little, P	2008	Randomised controlled trial of Alexander technique lessons, exercise, and massage (ATEAM) for chronic and recurrent back pain	463	No
Machado, L	2010	The effectiveness of the mckenzie method in addition to first-line care for acute low back pain: a randomized controlled trial	139	Yes
Mackawan, S	2007	Effects of traditional Thai massage versus joint mobilization on substance P and pain perception in patients with non-specific low back pain	68	Yes
Magnussen, L	2007	Motivating disability pensioners with back pain to return to work - A randomized controlled trial	89	No
Marshall, P	2008	Muscle Activation Changes After Exercise Rehabilitation for Chronic Low Back Pain	50	No
Marshall, P	2008	Self-Report Measures Best Explain Changes in Disability Compared With Physical Measures After Exercise Rehabilitation for Chronic Low Back Pain	50	Yes
Mazza, M	2010	Escitalopram 20 mg versus duloxetine 60 mg for the treatment of chronic low back pain	75	No
Mibielli, M	2009	Diclofenac plus B vitamins versus diclofenac monotherapy in lumbago: the DOLOR study	372	Yes
Mohseni-Bandpei, M	2006	A prospective randomised controlled trial of spinal manipulation and ultrasound in the treatment of chronic low back pain	112	No
Mohseni-Bandpei, M	2011	The effect of pelvic floor muscle exercise on women with chronic non-specific low back pain	20	Yes
Morone, G	2011	Quality of life improved by multidisciplinary back school program in patients with chronic non-specific low back pain: a single blind randomized controlled trial	70	Yes
Muehlbacher M	2006	Topiramate in Treatment of Patients With Chronic Low Back Pain	96	Yes
Muller-Schwefe, G	2011	Dysport(registered trademark) for the treatment of myofascial back pain: Results from an open-label, Phase II, randomized, multicenter, dose-ranging study	181	Yes
Muthukrishnan, R	2010	The differential effects of core stabilization exercise regime and conventional physiotherapy regime on postural control parameters during perturbation in patients with movement and control impairment chronic low back pain research	30	No
Nassif, H	2011	Evaluation of a randomized controlled trial in the management of chronic lower back pain in a french automotive industr: An Observational study	60	Yes

Lead author	Publication year	Title	Sample size	Extracted
Newcomer, K	2008	Is a videotape to change beliefs and behaviors superior to a standard videotape in acute low back pain? A randomized controlled trial	111	No
Nigg, B	2009	The Effectiveness of an Unstable Sandal on Low Back Pain and Golf Performance	37	No
Noredeman, L	2006	Early access to physical therapy treatment for subacute low back pain in primary health care: a prospective randomized clinical trial	60	Yes
Norris, C	2008	The role of an integrated back stability program in patients with chronic low back pain	59	Yes
Oleske, D	2007	Are Back Supports Plus Education More Effective Than Education Alone in Promoting Recovery From Low Back Pain?	433	No
Pach, D	2011	Efficacy of Injections with Disci/Rhus Toxicodendron Compositum for Chronic Low Back Pain - A Randomized Placebo-Controlled Trial	142	Yes
Paoloni, M	2011	Kinesio taping applied to lumbar muscles influences clinical and electromyographic characteristics in chronic low back patients.	39	Yes
Paolucci, T	2011	Psychological features and outcomes of the Back School treatment in patients with chronic non-specific low back pain. A randomized controlled study	50	No
Perez-Palomares, S	2010	Percutaneous Electrical Nerve Stimulation Versus Dry Needling: Effectiveness in the Treatment of Chronic Low Back Pain	124	No
Powers, C	2008	Effects of a single session of posterior-to-anterior spinal mobilization and press-up exercise on pain response and lumbar spine extension in people with nonspecific low back pain.	30	Yes
Rasmussen, J	2008	Manipulation does not add to the effect of extension exercises in chronic low-back pain (LBP). A randomized, controlled, double blind study	72	No
Rasmussen-Barr, E	2009	Graded Exercise for Recurrent Low-Back Pain A Randomized, Controlled Trial With 6-, 12-, and 36-Month Follow-ups	71	No
Ritvanen, T	2007	Dynamic surface electromyographic responses in chronic low back pain treated by traditional bone setting and conventional physical therapy	61	Yes
Roche, G	2007	Comparison of a functional restoration program with active individual physical therapy for patients with chronic low back pain: a randomized controlled trial	132	No
Roche-Leboucher, G	2011	Multidisciplinary Intensive Functional Restoration Versus Outpatient Active Physiotherapy in Chronic Low Back Pain	131	Yes

Lead author	Publication year	Title	Sample size	Extracted
Sahin, N	2011	Effectiveness of back school for treatment of pain and functional disability in patients with chronic low back pain: a randomized controlled trial	146	Yes
Santaella, K	2009	Assessment of a Biofeedback Program to Treat Chronic Low Back Pain	60	Yes
Schiltenwolf, M	2006	Comparison of a biopsychosocial therapy (BT) with a conventional biomedical therapy (MT) of subacute low back pain in the first episode of sick leave: a randomized controlled trial	61	Yes
Senna, M	2011	Does Maintained Spinal Manipulation Therapy for Chronic Nonspecific Low Back Pain Result in Better Long-Term Outcome?	88	Yes
Shankar, N	2011	Autonomic status and pain profile in patients of chronic low back pain and following electro acupuncture therapy: A randomized controlled trial	60	Yes
Sherman, K	2011	A Randomized Trial Comparing Yoga, Stretching, and a Self-care Book for Chronic Low Back Pain	206	Yes
Shirado, O	2010	Multicenter Randomized Controlled Trial to Evaluate the Effect of Home-Based Exercise on Patients With Chronic Low Back Pain	175	No
Skljarevski, C	2010	Efficacy and Safety of Duloxetine in Patients With Chronic Low Back Pain	236	No
Skljarevski, V	2009	Maintenance of Effect of Duloxetine in Patients with Chronic Low Back Pain: A 41-week Uncontrolled, Dose-blinded Study	177	Yes
Skljarevski, V	2009	A double-blind, randomized trial of duloxetine versus placebo in the management of chronic low back pain	404	No
Skljarevski, V	2010	Duloxetine Versus Placebo in Patients With Chronic Low Back Pain: A 12-Week, Fixed-Dose, Randomized, Double-Blind Trial	401	Yes
Sorensen, P	2010	An educational approach based on a non-injury model compared with individual symptom-based physical training in chronic LBP. A pragmatic, randomised trial with a one-year follow-up	185	Yes
Steenstra, I	2006	Economic Evaluation of a Multi-Stage Return to Work Program for Workers on Sick-Leave Due to Low Back Pain	196	No
Suen, L	2007	Auriculotherapy on low back pain in the elderly	60	Yes
Suni J	2006	Control of the Lumbar Neutral Zone Decreases Low Back Pain and Improves Self-Evaluated Work Ability	92	No
Szczurko, O	2007	Naturopathic Care for Chronic Low Back Pain: A Randomized Trial	59	No

Lead author	Publication year	Title	Sample size	Extracted
Tefner, I	2011	The effect of spa therapy in chronic low back pain: a randomized controlled, single-blind, follow-up study	37	Yes
Thomas K	2006	Randomized controlled trial of a short course of traditional acupuncture with usual care for persistent non-specific low back pain	215	Yes
Unsgaard-Tondel, M	2010	Motor Control Exercises, Sling Exercises, and General Exercises for Patients With Chronic Low Back Pain: A Randomized Controlled Trial With 1-Year Follow-up	109	Yes
van der Roer, N	2008	Intensive group training protocol versus guideline physiotherapy for patients with chronic low back pain: a randomised controlled trial	102	No
Vasseljen, O	2011	Effect of Core Stability Exercises on Feed-Forward Activation of Deep Abdominal Muscles in Chronic Low Back Pain	87	Yes
Vong, S	2011	Motivational Enhancement therapy in addition to physical therapy improves motivational factors and treatment outcomes in people with low back pain: A randomized controlled trial.	76	Yes
Weiner, D	2008	Efficacy of Percutaneous Electrical Nerve Stimulation and Therapeutic Exercise for Older Adults with Chronic Low Back Pain: A Randomized Controlled Trial	184	Yes
Wetherell, J L	2011	A randomized, controlled trial of acceptance and commitment therapy and cognitive-behavioral therapy for chronic pain	99	Yes
Whitfill, T	2010	Early Intervention Options for Acute Low Back Pain Patients: A Randomized Clinical Trial with One-Year Follow-Up Outcomes	102	Yes
Williams, K	2009	Evaluation of the Effectiveness and Efficacy of Iyengar Yoga Therapy on Chronic Low Back Pain	90	Yes
Witt, C	2006	Pragmatic randomized trial evaluating the clinical and economic effectiveness of Acupuncture for Chronic Low back Pain	2594	No
Yildirim, Y	2010	Relationship between learning strategies of patients and proper perception of the home exercise program with non-specific low back pain	26	No
Zaringhalam, J	2010	Reduction of chronic non-specific low back pain: A randomised controlled clinical trial on acupuncture and baclofen	80	Yes
